# Establishment of a donor pig for xenotransplantation clinical trials based on the principle of Changsha Communiqué

**DOI:** 10.1002/hcs2.37

**Published:** 2023-04-03

**Authors:** Xiaoqian Ma, Sang Li, Jia Wang, Chang Xu, Wei Wang

**Affiliations:** ^1^ Institute for Cell Transplantation and Gene Therapy The 3rd Xiangya Hospital of Central South University Changsha China; ^2^ Engineering and Technology Research Center for Xenotransplantation of Human Province Changsha China

**Keywords:** bio‐safety, donor pig, DPF, xenotransplantation

## Abstract

**Background:**

Xenotransplantation is a potential way to reduce the shortage of the needed organ grafts for the end‐stage disease. Immune rejection, physiological incompatibility and bio‐safety are the most critical issues.

**Methods:**

To ensure the safety and efficacy of gene editing, second‐ and third‐generation sequencing technologies have allowed us to obtain a clearer genetic background of donor pigs for xenotransplantation. Based on the Changsha Communiqué, the local DPF‐ excluded lists and DPF donor facility were established in Changsha, China. A pig‐to‐human islet clinical trial was conducted and overseen by the respective Chinese governmental agency.

**Results:**

The DPF standards for pig husbandry eliminated specific pathogens in donor pigs. We have established a PERV‐C free, genetic information clean, DPF donor for xenotransplantation. A clinical trial of ten adult patients (9M:1F) with type 1 diabetes who received DPF porcine islet xenotransplantation via the portal vein were performed. Clinical accepted immunosuppressant drugs and autologous Treg were used for controlling immune rejection. No cross‐species infection events occurred in this trial, and importantly, no cross‐species transmission of PERV was found.

**Conclusions:**

Xenotransplantation is a pioneer study and safety is the most important issue. The fundamental principles for establishing xenotransplantation donor pigs should follow the Changsha Communiqué (2008), the second WHO consultation,and the 2018 Changsha Communiqué which would finally help reducing the risks of xenotransplantation.

**Abbreviations**
CRISPR: Clustered Regularly Interspaced Short Palindromic Repeats
DPF: designated pathogen‐free
IXA: International Xenotransplantation Association
PERV: Porcine endogenous retrovirus
WHO: World Health Organization

Xenotransplantation is an attractive solution to bridge the gap between the demand for and availability of cadaveric human organs, tissues, and cells for clinical needs. The Changsha Communiqué released by the World Health Organization (WHO) is the first declaration outlining the international guideline for xenotransplantation clinical research [1]. However, before the clinical application of xenografts, three major hurdles must be overcome: immune rejection, physiological incompatibility, and the transmission of zoonoses. The donor animal is the most critical issue. The Changsha Communiqué declares that animals used in xenotransplantation should be acquired from a closed herd bred for the purpose of xenotransplantation and housed in a well‐controlled, pathogen‐free environment with high animal welfare standards. The donor animals should also be extensively tested to ensure freedom from known pathogens, with appropriate biosecurity and surveillance in place to ensure this.

Immune rejection and physiological incompatibility can perhaps be solved using gene‐editing technology. Genetic modifications of the donor pig can reduce the incidence of immune rejection and prolong the survival of porcine xenografts. Research achievements in this field have accelerated during the past decade because of novel gene‐editing and cloning technologies. Recently, Dr. Porrett [2] successfully performed kidney transplantation from a genetically engineered pig to a brain‐dead person (leaving the kidney outside the body) in Alabama. The kidney functioned immediately and was not rejected during the 74‐h period of the study. In early January 2022, Cardiac Xenotransplantation Program's clinical director Bartley P. Griffith and scientific/program director Muhammad Mohiuddin [3], successfully transplanted a gene‐edited pig heart into a patient with end‐stage cardiac failure at the University of Maryland School of Medicine. The donor pigs had 10 genetic modifications. Four genes were knocked out or inactivated, including one encoding a molecule (*N*‐glycolylneuraminic acid) that causes an aggressive human rejection response and a growth gene (growth hormone receptor) that was knocked out to prevent the pig's heart from growing after transplantation. In addition, six human genes were inserted into the genome of the donor pig to make the porcine organs more tolerable to the human immune system [4].

As described in the Changsha Communiqué (2008), genetic modification of the animals may improve the effectiveness of such xenotransplant material [1]. Developments in gene‐editing technology will undoubtedly help expedite porcine xenotransplantation. To ensure the safety and efficacy of gene editing, a clear understanding of its genetic background is important. The reference genome sequence is known to be important in biomedical studies using animal models [5]. Although the genomes of some pig breeds have been published, there is a paucity of chromosome‐level genomes with comprehensive sequence information. Moreover, most currently reported pig genomes are obtained from commercial pig breeds, which are unspecific for xenotransplantation. Second‐ and third‐generation sequencing technologies have allowed us to obtain a clearer genetic background of donor pigs for xenotransplantation.

The transmission of zoonoses is a main biosafety concern. Regarding monitoring of zoonotic infections, reference was made to the pathogen list published at the 2011 WHO Global Consultation Meeting in Geneva [6]. A curtailed list was proposed by Dr. Fishman, Gazda, Onions, and other experts also suggested several lists for what constitutes a designated pathogen [7, 8]. Recommendation 1 of the 2018 Changsha Communiqué which has been updated from “specific” to “designated” pathogen‐free animals to reflect current understanding of the conditions necessary to utilize specially bred animals for xenotransplantation [9]. The extensive lists of excluded agents defining the designated pathogen‐free (DPF) health status in the original consensus statement still serve as an appropriate basis to establish DPF criteria. Additional agents of uncertain clinical significance continue to be identified worldwide, for example, enterovirus B, rotaviruses, porcine teschovirus, *Paramyxoviridae* sp., Tioman virus, parvovirus PPARV‐4 (bocavirus), kobuvirus, novel astroviruses, and Ljungan‐like viruses. These evolving lists represent a global summary of agents. These DPF‐excluded agent lists should be dynamic and adaptive in response to new and emergent agents within the geography of the donor. Some examples illustrating the need for geography‐based monitoring and adaptive responses include the emergence of African swine fever in China in 2018 and porcine epidemic diarrhea in United States in 2013. These diseases have never infected humans [10, 11] but are of significant concern to pig health and production in affected areas. Stringent requirements to fulfill the DPF status applied to wild‐type pigs should be equally applied to genetically modified donor pigs [12].

The DPF standards for pig husbandry can help eliminate specific pathogens in donor pigs. Porcine endogenous retroviruses (PERVs) are present in the genome of all pig strains. PERV are divided into three classes, PERV‐A, PERV‐B, and PERV‐C. The recombinant PERV‐A/C can infect human cells in vitro with greater efficiency. Questions are still being asked about the relevance of PERV as a pathogen and the risk of unknown elements not detected in the initial screening of both animal and product. These are difficult to answer; in essence, to fully evaluate the microbiological risk, clinical trials are needed [9]. WHO and International Xenotransplantation Association (IXA) have proposed that a PERV‐C‐free genome is recommended in donor pigs for xenotransplantation [9, 13].

In 1999, Wang et al. screened 11 local pig breeds in China and selected a breed that lacked a high proportion of PERV‐C. Following its identification, a pig line negative for PERV‐C was established and inbred for 22 generations (Figure 1). A barrier facility for DPF pigs was constructed in Changsha, with operations starting in 2012 [9]. This facility has the infrastructure and equipment to breed and maintain animals in DPF conditions in compliance with the regulations in China. Dr. Wang illustrated this with a list of pathogens excluded from the herd with stringent ongoing maintenance and conditions, such as specialized feeding. Accordingly, the porcine herd Xeno‐1 was selected as a potential donor for xenotransplantation because of its natural PERV‐C‐free status [14]. In 2013, a pig‐to‐human islet clinical trial overseen by the respective Chinese governmental agency was conducted by Dr. Wang; the design of this trial regarding biosafety monitoring was outlined in detail. This trial included the implementation of a database for clinical trials and the archiving of medical records. The trial was also registered at ClinicalTrials. Gov (NCT03162237) (Figure 2). Based on current WHO guidelines and existing pathogen list for DPF, Dr. Wang designed Hunan local DPF pathogen list (Table 1). They conducted a clinical trial of 10 adult patients (9M:1F) with type 1 diabetes who received DPF porcine islet xenotransplantation via the portal vein. After islet infusion, distal end of the catheter was kept in portal vein, and proximal of the catheter was connected with a drug perfusion pump for anticoagulant therapy for 7 days to control instant blood mediated inflammatory reaction. Clinical accepted immunosuppressant drugs and autologous Treg were used for controlling immune rejection. No cross‐species infection events occurred in this trial, and importantly, no cross‐species transmission of PERV was found (the trial is still ongoing, and results are not published). Similar results were reported in the other two clinical trials on islet xenotransplantation [15, 16].

**Figure 1:**
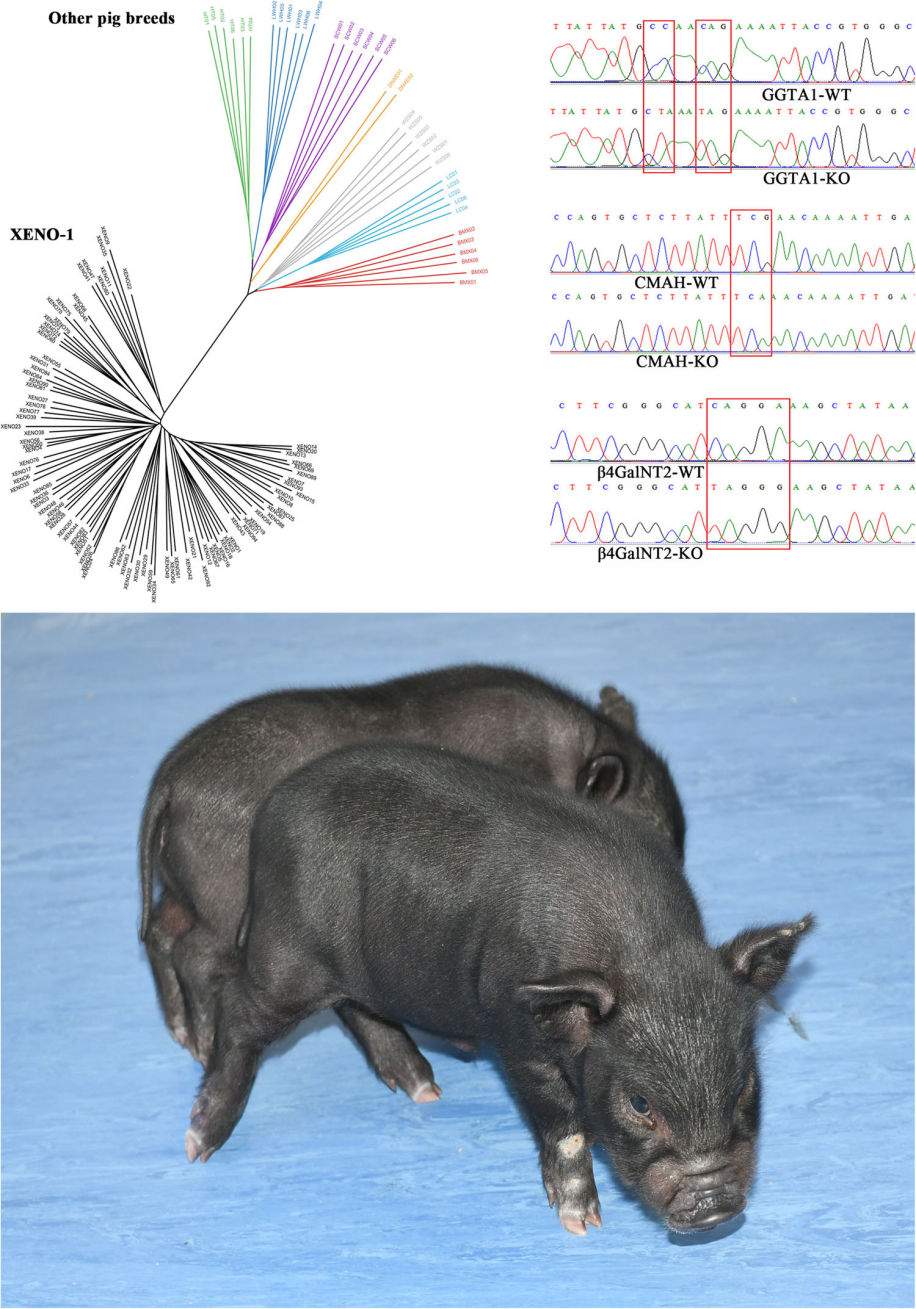
Genetically modified DPF donor pigs for xenotransplantation. The phylogenetic tree analysis for Xeno‐1 pigs (left top). The DNA sequencing for GGTA1‐KO/CAMH‐KO/β4GalNT2‐KO transgenic Xeno pigs (right top). The DPF Xeno pigs (down). DPF, designated pathogen‐free.

**Figure 2:**
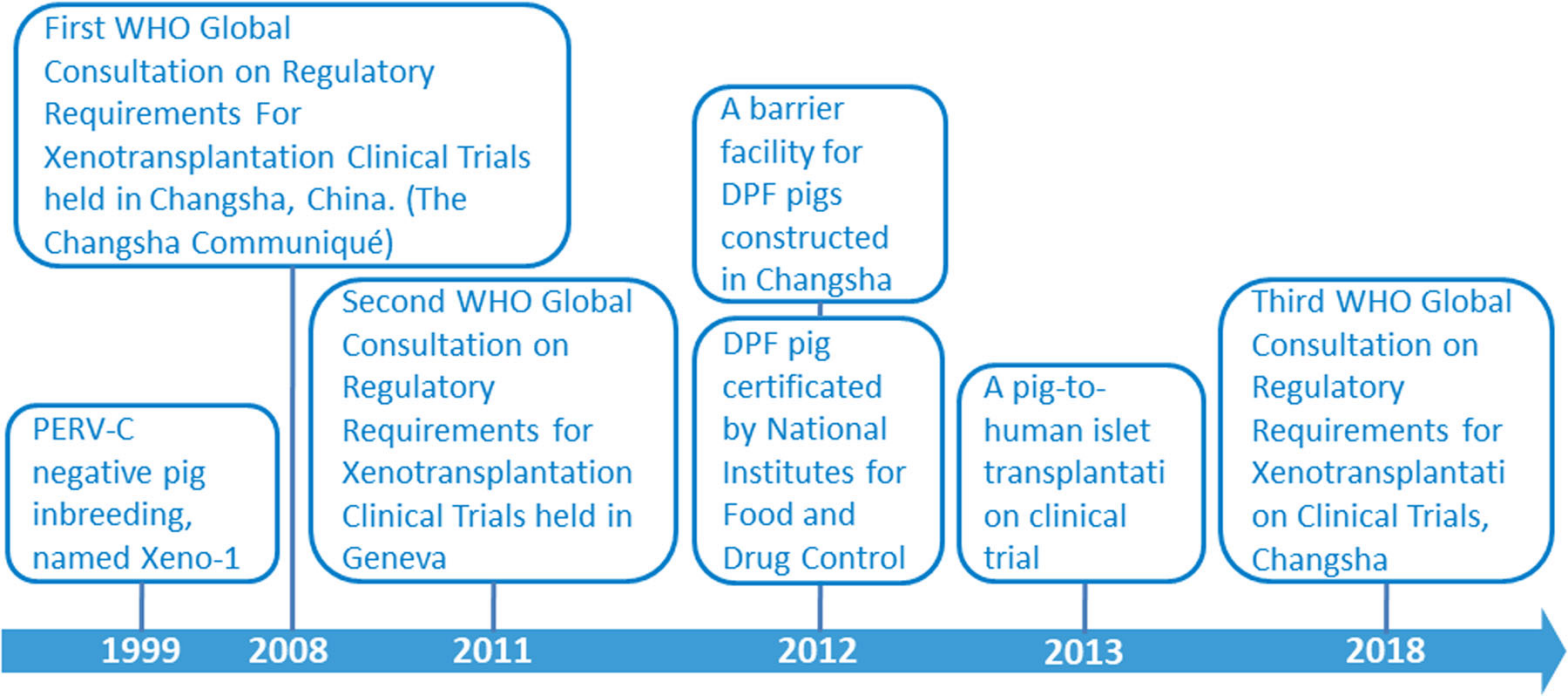
The schematic illustration of our work in xenotransplantation followed by the WHO consultation on regulatory requirements for xenotransplantation on clinical trials.

**Table 1:** The designated pathogen list.

**Bacteria**
*Brucella* spp.
*Leptospira* spp.
*Serpulina hyodysenteriae*
*Mycobacterium bovis*
*Mycobacterium tuberculosis*
*Mycobacterium avium*‐intracelluare complex
*Mycoplasma hyopneumoniae*
*Salmonella typhi*
*Shigella*
*Bordetella bronchiseptica*
*Pasteurella multocida*
*Actinobacillus pleuropneumoniae*
*Streptococcus suis* type 2
**Fungi**
Pathogenic dermal fungi
*Cryptococcus neoforman*
*Histoplasma capsulatum*
**Parasites**
Ectozoa
*Ascaris suum*
*Echinococcus* sp.
*Isospora* sp.
*Strongyloides ransomi*
*Toxoplasma gondii*
*Trichinella spiralis*
*Neospora*
*Fasciolopsis buski*
**Viruses**
Adenovirus (porcine)
Encephalomyocarditis virus
Porcine influenza virus
Human influenza viruses
Porcine cytomegalovirus
Porcine gama‐herpesvirus
Porcine reproductive and respiratory syndrome virus
Procine parvovirus
Rotavirus
Pseudorables virus
Rabies virus
Foot and mouth disease virus
Classical swine fever virus
Japaneses encephalitis virus
Porcine circovirus type 2
Porcine transmissible gastroenteritis virus
Swine vesicular disease virus
Porcine endogenous retrovirus (PERV)
Porcine lymphotropic herpesvirus (PLHV)
Hepatitis E virus (HEV)
Porcine circovirus 1

Xenotransplantation is a pioneer study, and safety is the most important issue. Various tumors are well known to show increased incidence in organ allotransplant recipients as they have been receiving longterm immunosuppressive therapy [17, 18, 19]. Whether this effect would to be the case after xenotransplantation has not yet been well investigated because of the small number of recipients. Moreover xenograft would live in human recipient for a long time, the risk of graft malignancy will be definitely increased because of age [20]. Therefore, more attention should be paid to the carcinogenesis related genes screening in donor pigs.

The rapid development of pig genetic engineering, particularly with the introduction of genome‐editing techniques, such as clustered regulary interspaced short palindromic repeats‐Cas, has advanced the development of xenotransplantation research. Biosafety is still the primary issue in clinical trials. We believe that the fundamental principles for establishing xenotransplantation donor pigs should follow the Changsha Communiqué (2008) [1, 21], the second WHO consultation [13], and the 2018 Changsha Communiqué [9].

## AUTHOR CONTRIBUTIONS

**Xiaoqian Ma**: Writing—original draft (equal). **Sang Li**: Writing—original draft (equal). **Jia Wang**: Data curation (equal). **Chang Xu**: Data curation (equal). **Wei Wang**: Supervision (equal); Writing—review and editing (equal).

## CONFLICT OF INTEREST STATEMENT

The authors declare no conflict of interest.

## ETHICS STATEMENT

This study was approved by Hunan Department of Health. Each recipient was evaluated and approved by the Ethics Committee of Xiangya Third Hospital (Changsha, China) case by case and was registered with ClinicalTrials.gov (NCT03162237).

## INFORMED CONSENT

All recipients provided their written consent to participate in this study. Ethical approval was renewed on an annual basis.

## Data Availability

The data that support the findings of this study are available from the corresponding author upon reasonable request.
